# Development of Standards for Presenting and Reporting clinical InterveNtions Televisually (SPRINT) reporting guidelines: A study protocol

**DOI:** 10.1371/journal.pone.0351633

**Published:** 2026-07-09

**Authors:** Michael G. Fadel, Henry Douglas Robb, Bibek Das, Ayda Alizadeh, Aksaan Arif, Olivia Ariarasa, Matyas Fehervari, Hutan Ashrafian

**Affiliations:** 1 Department of Surgery and Cancer, Imperial College London, London, United Kingdom; 2 Department of General Surgery, Chelsea and Westminster Hospital, London, United Kingdom; 3 Department of Upper Gastrointestinal Surgery, Hammersmith Hospital, London, United Kingdom; 4 Faculty of Medicine, Imperial College London, London, United Kingdom; 5 Bariatric Surgery Department, Maidstone and Tunbridge Wells NHS Trust, Kent, United Kingdom; Tribhuvan University Institute of Medicine, NEPAL

## Abstract

**Background:**

Videos of clinical intervention (VoCI) demonstrate medical and surgical approaches, including laparoscopy and robotics, endoscopy and interventional techniques. VoCI allow viewers to easily understand the steps of a specific procedure, enhancing clinical training through the acquisition of key perceptual and cognitive skills. However, there can be heterogeneity in video reporting, leading to the omission of critical procedural steps and suboptimal visual quality. This protocol describes the development of Standards for Presenting and Reporting clinical InterveNtions Televisually (SPRINT) guidelines through a Delphi process, which will provide a set of minimum items that should be reported by clinicians when uploading VoCI to improve their consistency and completeness.

**Methods and analysis:**

The development of SPRINT reporting guidelines will follow five key steps. In step 1, a Steering Committee has been established to lead and coordinate the consensus development process. In step 2, a scoping review of current VoCI has been performed to generate preliminary checklist items. In step 3, a diverse international panel of clinical experts, trainees, educationalists, media platform providers and patient representatives will participate in a Delphi process, seeking ≥ 70% agreement on checklist items. In step 4, the SPRINT reporting guidelines will be finalised, along with an explanation and elaboration (E&E) document. In step 5, the guidelines will be broadly disseminated to ensure effective implementation.

**Registration details:**

The project is pre-registered within the Open Science Framework (https://osf.io/an2jt/) and the Enhancing the QUAlity and Transparency Of health Research (EQUATOR) Network.

## Introduction

Videos of clinical intervention (VoCI) are becoming increasingly important in surgical and medical education, demonstrating surgical (e.g., laparoscopic and robotic), endoscopic, interventional and radiological procedures. VoCI provide the opportunity to learn new techniques, view rare cases and allow residents to access videos internationally [1–4]. They play an essential role in simulation training, measuring quality and monitoring progression, helping to enhance clinical training and skill development [5–9]. VoCI also promotes patient empowerment and education through raising awareness about specific conditions and providing insights into their experience [10–14]. Video recordings allow for retrospective intraoperative analysis to identify any potential causes of complications [3], and can provide objective evidence in medicolegal cases.

The application of VoCI in clinical, educational and academic settings has the potential to benefit clinical practice and patient outcomes. Online educational peer-reviewed resources such as WebSurg (Research Institute against Cancer of the Digestive System), provides expert content that meets the Health on the Net Foundation Code of Conduct requirements for quality, confidentiality and transparency [15,16]. These readily available videos are becoming an essential component of surgical education and can be presented in various methods, including academic journals, educational resources (e.g., Proximie, Touch Surgery) and commercial platforms (e.g., YouTube) [7,17]. However, media platforms can present videos in variable forms and heterogenous reporting of videos, which may reduce the quality, reliability and educational value of the intended learning outcome. They also have the potential to contain incorrect promotional information [18,19].

There is currently no established evidence base on the application of televisual or presentation tools for craft specialties in clinical practice. Additionally, there is no established consensus or guidelines amongst international societies regarding the reporting of VoCI. To help address this, we aim to develop Standards for Presenting and Reporting clinical InterveNtions Televisually (SPRINT) for VoCI reporting to ensure high quality and technically accurate published videos. This in turn should lead to a more complete and transparent reporting of videos by health researchers, thus facilitating evidence-based decision making and reproducible research. This protocol describes the development of SPRINT reporting guidelines through a Delphi process, intended to provide a set of minimum items that should be reported by clinicians when uploading VoCI.

## Materials and methods

**Fig 1:**
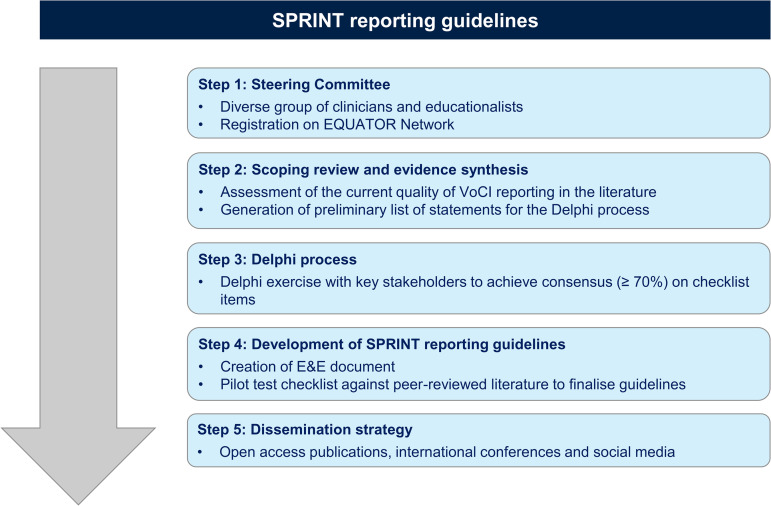
The five key steps to develop Standards for Presenting and Reporting clinical InterveNtions Televisually (SPRINT) reporting guidelines. E&E, explanation and elaboration; EQUATOR, Enhancing the QUAlity and Transparency Of health Research; VoCI, video of clinical intervention.

SPRINT guidelines will be developed through five key steps (Fig 1), which will follow the Accurate Consensus Reporting Document (ACCORD) guidelines [20]. The study protocol has been pre-registered within the Open Science Framework [21] and the Enhancing the QUAlity and Transparency Of health Research (EQUATOR) Network registry for reporting guidelines [22]. The study received ethical approval in May 2024 and the scoping review of video-based literature has been completed. The results of the review have been used to generate the preliminary checklist items for the Delphi process. Participant recruitment and data collection for the Delphi process will commence in April 2026 and is expected to be completed by November 2026. The finalisation of the SPRINT guidelines and production of the explanation and elaboration (E&E) is expected to be carried out by December 2026, followed by the dissemination and implementation strategy. The overall study is anticipated to be completed by April 2027.

### Step 1: Steering Committee

We have formed a Steering Committee [MGF, HDR, BD, AA, AA, OA, MF, HA] to oversee the guideline development process of SPRINT. The committee members are a diverse group of clinicians and educationalists, and have experience in developing reporting guidelines. MGF is leading the development of START-EDI (STAndards for ReporTing Equality, Diversity and Inclusion) [23,24] and HA has vast experience and expertise in the development of reporting guideline initiatives such as STARD-AI (Standards for Reporting of Diagnostic Accuracy Study-Artificial Intelligence) [25] and QUADAS-AI (QUality Assessment of Diagnostic Accuracy Studies-Artificial Intelligence) [26].

### Step 2: Scoping review and evidence synthesis

A scoping review was performed to investigate the evidence gap and current quality in reporting VoCI [27]. This was conducted according to the Preferred Reporting Items for Systematic Reviews and Meta-Analyses – Extension for Scoping Reviews (PRISMA-ScR) guidelines [28].

#### Search strategy and data extraction.

A literature search of MEDLINE, Embase, Emcare and CINAHL databases was performed. Search strategies were formulated using Medical Subject Headings (MeSH) terms such as video, minimal access surgery, laparoscopy, robotic surgery, endoscopy, radiology, interventional, training and education. Published articles containing VoCI, since January 2020, were retrieved. The full search strategy and study selection process is described in Robb et al. [27].

Data was extracted from the identified articles according to the following three domains:

1. **Video characteristics** – first author, year, country, specialty, procedure, video format and length, case presentation and outcomes, presence of audio/text commentary, disclosures and subtitles.
2. **Video utility** – thematic analysis on the purpose of the video, the intended target audience and the reproducibility of demonstrated skill or procedure.
3. **Video quality** – subjective and objective measurements of the quality of the video.

Following the review of the literature, the essential components of VoCI reporting have been defined to generate the preliminary list of checklist items for the Delphi process described below.

### Step 3: Delphi process

#### Delphi panel.

The Delphi methodology is a widely accepted technique for reaching a consensus among a panel of experts [29]. We will invite at least 50–60 participants as a diverse group of surgical and medical experts and trainees with experience in televisual training. We endeavour to have at least two participants from each of the subgroups of specialities to allow us to develop a universal checklist for VoCI reporting. These include but not limited to: surgical specialities (e.g., General, Urology, Vascular, Plastic, Cardiothoracic, Ear, Nose and Throat, Neurosurgery, Orthopaedics, Obstetrics and Gynaecology), medical interventionalists (e.g., Gastroenterology, Respiratory, Cardiology and Dermatology), radiology and interventional radiology and media platform representatives (e.g., WebSurg, Touch Surgery, Proximie, C-SATS and Incision). Other groups of stakeholders will also be involved during this process, including policy/regulatory agencies, guideline developers, representatives of organisations advocating transparency in research (e.g., EQUATOR), and patient and public representatives. The patient and public representatives will help bring a valuable perspective to the discussion regarding VoCI reporting and will help promote transparency.

#### Consensus development.

The preliminary list of checklist items agreed on by the Steering Committee will be assigned to the following categories: (1) educational content and useability; (2) case presentation, critical steps of the procedure and patient outcomes; (3) video quality, length and speed; (4) supportive information and commentary and (5) validation and ethics. For each item, the Delphi panel will provide their responses using a Grading of Recommendations Assessment, Development and Evaluations (GRADE) 9-point scale [24,30–34] via an electronic voting platform (Qualtrics XM [35]):

- 1–3 = of limited importance (item should not be included in the reporting guidelines);
- 4–6 = important but not critical (item should be discussed in the consensus meeting);
- 7–9 = important and critical (item should be included in the reporting guidelines).

We will also include a “do not know” option, for participants who do not feel qualified to rank any specific item. Free-text fields will be provided to allow panellists to suggest modifications or additional items.

There will be two rounds of online voting, where the responses will be analysed and summarised by the Steering Committee between each round. We will use the following established consensus definitions [24,30,33]:

- Consensus for inclusion: ≥ 70% participants scoring 7–9 and <15% participants scoring 1–3;
- Consensus for exclusion: ≥ 70% participants scoring 1–3 and <15% participants scoring 7–9;
- No consensus for inclusion or exclusion: failure to achieve either of the above.

Items that do not achieve consensus in rounds one or two will be reviewed, and these will be revised or eliminated considering any free-text comments.

The two rounds of voting will be followed by a hybrid consensus meeting in order to agree on the final items for inclusion in SPRINT reporting guidelines. The Steering Committee will select approximately 15–20 participants from those interested based on; (i) their ability to attend the meeting on proposed dates and times and (ii) the need to have an international multidisciplinary group of stakeholders [33,36]. Steering Committee members will also be present during the meeting. Dissemination and implementation strategies will also be discussed during the meeting.

### Step 4: Development of SPRINT reporting guidelines

On completion of the Delphi process, the checklist items for reporting VoCI will be finalised by the Steering Committee. A separate E&E document will provide a detailed rationale for the items included in the checklist. To assess usability, the reporting checklist will be pilot tested [37,38] against video peer-reviewed literature of several standardised operations in the first instance, such as laparoscopic and robotic total mesorectal excision, sleeve gastrectomy and endoscopic procedures.

### Step 5: Dissemination strategy

The development of SPRINT guidelines will be published in an open access format, and a SPRINT checklist and flow diagram will be freely available. We will disseminate the guidelines through social media channels, such as LinkedIn and X, and through media platform providers. The SPRINT guidelines will be presented at international conferences, such as the World Conferences on Research Integrity (WCRI) and the European Association for Endoscopic Surgery (EAES) conference.

A dedicated group website [39] has been created, which will contain additional resources and webinars for healthcare professionals and the public. We will be in contact with editors of leading journals and seek endorsement from groups of medical and surgical editors such as the International Committee of Medical Journal Editors and Surgery Journal Editors Group. We will also provide updated entries on the EQUATOR Network database and disseminate SPRINT through their promotional activities. We plan to later evaluate the impact of the reporting guidance through a systematic review and measure web metrics to determine the number of associated citations, and update the guidelines as necessary.

### Ethical approval

The study has obtained ethical approval (study ID: 6848347) from the Imperial College Research Ethics Committee (ICREC). Written informed consent and conflict of interest forms will be obtained from all participants during the Delphi process.

## Discussion

The applications of VoCI are broad and variable, having a strong impact on clinical practice and patient outcome. Intraoperative video-based technical skills assessment, analysed in a systematic review by Balvardi et al. [40], supported the association between superior surgical techniques and lower postoperative morbidity. The authors proposed video analysis as an approach to surgical quality improvement. A pilot study on the educational quality of laparoscopic colorectal surgery videos highlighted the need for a standardised approach for reporting of educational videos in laparoscopic colorectal surgery [41], which led to the development of LAP-VEGaS (LAParoscopic surgery Video Educational GuidelineS) [42]. The majority of videos available on the internet were found to be deficient in many aspects, including a lack of information on patients’ data such as age, body mass index, history of previous surgery, indication for surgery, and surgical outcomes such as morbidity and histopathological assessment. Through the development of these guidelines, a survey among surgical trainees also suggested that in order for a laparoscopic video to be a useful educational tool, it should contain diagrams, snapshots, tables and audio-commentary. A further study, performed by co-authors of this manuscript, found that published laparoscopic sleeve gastrectomy videos, along with other surgical videos, can fail to report all the critical steps of the procedure and patient outcomes in the short-term, due to a lack of quality control and a standardised checklist [27,43].

**Fig 2:**
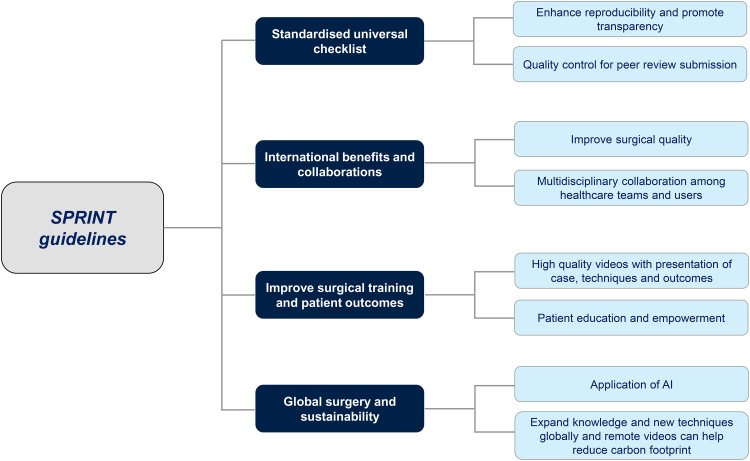
The anticipated benefits and outcomes of SPRINT reporting guidelines. AI, artificial intelligence.

SPRINT will be developed through a comprehensive five-step process to provide an universal reporting guidelines for video-based clinical procedures (including surgery, endoscopy and interventional radiology). The checklist can be adopted by several specialties and stakeholders including authors, peer reviewers and journal editors. SPRINT should help improve the completeness and transparency of video reporting (Fig 2). It should lead to an enhancement in surgical quality and an improvement in videos should enhance patient empowerment and awareness of the steps of the procedure. Videos are of low/no cost and higher quality educational videos will also have the benefit of contributing surgical knowledge and new techniques to different areas and regions globally, which may indirectly have the added benefit of reducing the carbon footprint.

During the development of the proposed SPRINT guidelines, artificial intelligence (AI) may have an important role to play. The increasing number of operative videos provides a rich global dataset for AI algorithms and therefore it is essential that these videos are of the highest possible standard. Machine learning applied to surgical videos offers the opportunity to clarify anatomy, detail the steps in a procedure, and provide real-time feedback and guidance [44–47]. Trainees are also able to use large language models, such as ChatGPT, to easily access educational video resources in a user-friendly format to improve their knowledge base. As a result, with the role of large language models having visual generating and appraisal activity, the volume of these teaching tools is increasing and so there is an even higher urgency to produce a set of standards in the form of SPRINT. The guidelines will provide a learning checklist and framework to train AI. Machine learning will be able to apply the guidelines to make predictions about data which will automatically improve over time, for example to guide AI to report on videos or guide the review process for submitted manuscripts. In addition, the regulations of AI software as medical devices are currently not yet fully clarified. The SPRINT guidelines can guide AI quality control as a teaching tool and help increase surgical safety and mitigate regulatory issues.

## Conclusions

In summary, SPRINT can help standardise the reporting of VoCI to ensure that the content of published videos is of the highest quality and accuracy. It will allow enhanced categorisation of VoCI for application of digital and AI-training and education tools, which may potentially improve usability and applicability of VoCI in future clinical training. SPRINT will also help provide a universal checklist for reviewing videos submitted for publication or conference presentation enhancing reproducibility and promoting greater transparency. We therefore anticipate that SPRINT will benefit clinicians, authors, editors, peer reviewers, guideline developers, members of the public, healthcare providers, and policymakers.
